# Modulation of Fatty Acid-Related Genes in the Response of H9c2 Cardiac Cells to Palmitate and n-3 Polyunsaturated Fatty Acids

**DOI:** 10.3390/cells9030537

**Published:** 2020-02-26

**Authors:** Silvia Cetrullo, Stefania D’Adamo, Veronica Panichi, Rosa Maria Borzì, Carla Pignatti, Flavio Flamigni

**Affiliations:** 1 Dipartimento di Scienze Biomediche e Neuromotorie, Università di Bologna, 40138 Bologna, Italy; 2 Dipartimento di Scienze Mediche e Chirurgiche, Università di Bologna, 40138 Bologna, Italy; 3 Laboratorio di Immunoreumatologia e Rigenerazione Tissutale, IRCCS Istituto Ortopedico Rizzoli, 40136 Bologna, Italy; rosamaria.borzi@ior.it

**Keywords:** eicosapentaenoic acid, docosahexaenoic acid, palmitic acid, nutraceuticals, miR-33, apoptosis, hypertrophy, H9c2 cardiomyoblasts

## Abstract

While high levels of saturated fatty acids are associated with impairment of cardiovascular functions, n-3 polyunsaturated fatty acids (PUFAs) have been shown to exert protective effects. However the molecular mechanisms underlying this evidence are not completely understood. In the present study we have used rat H9c2 ventricular cardiomyoblasts as a cellular model of lipotoxicity to highlight the effects of palmitate, a saturated fatty acid, on genetic and epigenetic modulation of fatty acid metabolism and fate, and the ability of PUFAs, eicosapentaenoic acid, and docosahexaenoic acid, to contrast the actions that may contribute to cardiac dysfunction and remodeling. Treatment with a high dose of palmitate provoked mitochondrial depolarization, apoptosis, and hypertrophy of cardiomyoblasts. Palmitate also enhanced the mRNA levels of sterol regulatory element-binding proteins (SREBPs), a family of master transcription factors for lipogenesis, and it favored the expression of genes encoding key enzymes that metabolically activate palmitate and commit it to biosynthetic pathways. Moreover, miR-33a, a highly conserved microRNA embedded in an intronic sequence of the SREBP2 gene, was co-expressed with the SREBP2 messenger, while its target carnitine palmitoyltransferase-1b was down-regulated. Manipulation of the levels of miR-33a and SREBPs allowed us to understand their involvement in cell death and hypertrophy. The simultaneous addition of PUFAs prevented the effects of palmitate and protected H9c2 cells. These results may have implications for the control of cardiac metabolism and dysfunction, particularly in relation to dietary habits and the quality of fatty acid intake.

## 1. Introduction

Although it is noteworthy that high-lipid diets have a negative impact on health state, increasing evidence supports the view that fatty acids (FAs), derived from different foods, do not have the same biological effects. Excessive levels of dietary saturated FAs or an imbalance of saturated versus unsaturated fats have been implicated in several age-related pathological conditions, such as cardiovascular diseases [1].

High levels of saturated FAs, such as palmitic acid (C16:0) and stearic acid (C18:0), have been shown to favor cell death, hypertrophy and insulin resistance in a variety of cell types, including cardiac cells, both in vivo and in vitro [2,3,4]. Several mechanisms have been suggested to contribute to the cytotoxicity and dysfunction of cells treated with high concentrations of palmitate, implicating in particular the metabolic fate of this compound. A hypothesis relies on the de novo synthesis of ceramide [5]; other postulated mechanisms of lipotoxicity include accumulation of diacylglycerol (DAG) and other metabolic intermediates, due to the inability of the cell to handle high concentrations of palmitate [6,7].

Long-chain FAs and, among these, saturated FAs, are the main source of energy for the heart and their oxidation requires the entry into the mitochondrion by the carnitine shuttle. However, FAs can also have other relevant fates in cells following their activation to acyl-CoA, such as glycerophospholipid and sphingolipid synthesis or, even in a non-adipose tissue like cardiac muscle, triglyceride (TG) synthesis, causing ectopic deposition of fat. On the one hand, this ectopic TG accumulation in cells that do not typically store fat, may alter cellular physiology contributing to heart dysfunction, on the other hand, however, it may represent a detoxification strategy to decrease free FAs [8].

Sterol regulatory element-binding proteins (SREBPs) are major transcription factors involved in the regulation of cholesterol- and FA-biosynthesis and fate, by modulating the expression of genes coding for crucial enzymes [9,10,11,12]. Besides, miR-33a, a highly conserved microRNA harbored in an intron of the SREBP2 gene, is co-expressed with the SREBP2 messenger and cooperates with the regulation of genes involved in lipid and FA metabolism [13]. In particular, targets of miR-33a include carnitine palmitoyl-transferase 1 (CPT1) and other enzymes that mediate the entry of long-chain fatty acids into the mitochondrial matrix and their subsequent β-oxidation [14,15]. Human beings express a second member of the miR-33 family encoded by intron 17 of the SREBP1 gene, named miR-33b, which however has a seed sequence identical to that of miR-33a and overlapping mRNAs targets.

In contrast to saturated FAs in excess, several studies have revealed a range of biological properties of n-3 polyunsaturated fatty acids (PUFAs), thus suggesting beneficial effects in the prevention or treatment of chronic and degenerative diseases, especially cardiovascular diseases [16,17]. n-3 PUFAs include α-linolenic acid (ALA, C18:3n-3), eicosapentaenoic acid (EPA, C20:5n-3), and docosahexaenoic acid (DHA, C22:6n-3), which have overlapping but also distinct properties [18]. Traditional sources of EPA and DHA (oily fish and fish oil capsules) also contain some n-3 docosapentaenoic acid (DPA, C22:5n-3), but its levels are very low compared to those of the former two. DPA is also considered a source of EPA (by retro-conversion) in vivo and, to a lesser extent, of DHA, thus implying molecular effects potentially associated to these FAs [19]. We have previously demonstrated that the long chain n-3 PUFA, EPA, is able to prevent the apoptotic death of H9c2 cardiac cells caused by excessive palmitate, even when added to the cell medium at a much lower dosage than palmitate [20]. However, the mode by which EPA interferes with palmitate-induced cell death and dysfunction remained unclear.

In the present study we describe some effects of palmitate on genetic and epigenetic regulation of key factors controlling FA metabolism and fate in H9c2 cells, and the ability of long-chain n-3 PUFAs, EPA and DHA, to contrast these actions that may contribute to cardiac dysfunction and remodeling. This research increases our knowledge of molecular mechanisms underlying the nutraceutical value of PUFAs, thus substantiating their supplementation in counteracting cardiovascular damage occurring after excessive saturated FA intake.

## 2. Materials and Methods

### 2.1. Cell Culture and Treatments

Rat H9c2 cardiomyoblast cells were cultured in Dulbecco’s modified Eagle’s medium (DMEM) supplemented with 10% fetal bovine serum (FBS), 100 IU/mL penicillin, 0.1 µg/mL streptomycin, and 1 mM l-glutamine for 48 h before treatments. FA supplementation was performed as previously described [21]. Palmitate 5 mM stock solution was prepared with 10% fatty acid-free albumin from bovine serum (BSA) and 5% ethanol. Stock solutions (10 mM EPA and DHA) were in absolute ethanol. Medium supplementation with 30 µM BSA, 60 µM EPA, and 60 µM DHA was simultaneous with 500 µM palmitate treatment. Control culture was treated with the same solutions without FAs. After 16 or 24 h of treatment, the cells were collected for following analysis.

### 2.2. Cell Viability and Caspase Activity

Cell viability was assessed by trypan blue exclusion test. Dead cells (including the dye) were counted and reported as a percentage of the total number of cells. Caspase activity was measured by the cleavage of the fluorogenic peptide substrate Ac-Asp-Glu-Val-Asp-7-amido-4-methylcoumarin (Ac-DEVD-AMC) as previously described [22] with minor changes. This compound is a substrate for caspase 3 and other effector caspases. Briefly, the cells were harvested in RIPA Lysis and Extraction Buffer (Thermo Fisher Scientific), sonicated, centrifuged for 10 min at 14,000 g at 4 °C and the supernatant used to assay enzyme activity. Protein extracts were combined with the assay buffer containing 100 mM Hepes, pH 7; 5 mM dithiothreitol, 0.1% (*v*/*v*); CHAPS, 10% (*w*/*v*); sucrose; and 150 µM Ac-DEVD-AMC and incubated for 15 min at 37 °C. The reaction was stopped in ice by adding 2% (*w*/*v*) sodium acetate in 0.2 M acetic acid. Finally, 7-amino-4-methylcoumarin (AMC) liberation was measured after dilution of samples and by using excitation at 370 nm and emission at 455 nm.

### 2.3. Mitochondrial Potential Assay

Mitochondrial potential was assessed by using Muse MitoPotential Kit (Merck Millipore, Burlington, MA, USA) according to the user’s guide in order to determine the percentages of cells exhibiting a change in mitochondrial polarization. The assay utilizes a cationic, lipophilic dye to detect changes in the mitochondrial membrane potential. The results were obtained using the Muse™ Cell Analyzer (Merck Millipore, Burlington, MA, USA).

### 2.4. Cell Size And Lipid Content Measurement

The effects of different treatments on cardiomyoblasts with regard to cell size and lipid content were analyzed exploiting flow cytometric detection with a FACSCanto II (Becton Dickinson, Franklin Lakes, NJ, USA). Briefly, at the end of treatments, cells were collected by trypsinization and, after centrifugation, the pelleted cells were gently resuspended and fixed with 2% paraformaldehyde for 10 min at room temperature. Then the cells were pelleted at 1000× *g*, resuspended in PBS and kept at 4 °C until the time of processing. At the time of lipid content analysis, volumes of cell suspensions containing 100,000 fixed cells were spun at 1000× *g* after the addition of 100 µL 0.1% Tween in TBS, resuspended with 200 µL 20 mM TBS pH 7.6, and stained with Nile red (10 μg/mL) for 2 h [23]. Then the samples underwent flow cytometric analysis—Nile red was excited with a 488 nm laser and fluorescent emission signals were collected at 575 nm wavelength. The measurement of forward scatter (FSC) allowed us to discriminate the cell size. For each sample, several thousand cells were analyzed, and different samples were compared taking into account the median channel of fluorescence intensity of the cells.

For qualitative evaluation of lipid content, cell monolayers were stained with Nile red and observed with an IX-50 Olympus inverted microscope with a TRITC filter set.

### 2.5. Real-Time RT-PCR

Total cellular RNAs were extracted with TRIzol (Invitrogen), according to manufacturer’s instructions. Total RNA (100 ng) was reverse-transcribed by using random primers and the reagents provided with the SuperScript VILO System for RT-PCR (Invitrogen). Real Time PCR analyses were performed by means of the QuantiTect SYBR Green PCR kit (TaKaRa) according to the following protocol: activation of HotStart Taq DNA polymerase at 95 °C for 10 sec, amplification (40 cycles: 95 °C for 5 sec followed by 58 °C for 20 sec). The amount of mRNA was normalized to glyceraldehyde 3-phosphate dehydrogenase (GAPDH) expression in each sample and referred to the control sample. The sequences of primers (from Invitrogen) are shown in Table 1. Finally, melting curves were evaluated to check the specificity of the primers. Gene expression levels were calculated by the ΔΔ cycle threshold (Ct) method.

MicroRNAs were reverse-transcripted by TaqMan MicroRNA RT kit (Life Technologies) and qPCR was performed with TaqMan Universal Mastermix (Life Technologies) following kit instructions. Mature miR quantification was performed using TaqMan MicroRNA Assays for miR-33a and U6 snRNA (internal control), according to manufacturer’s recommended protocols (Applied Biosystems) and as previously detailed [24].

### 2.6. Western Blotting

The cells were harvested in RIPA Lysis and Extraction Buffer (Thermo Fisher Scientific, Waltham, MA, USA), sonicated, centrifuged for 10 min at 14,000 g at 4 °C and the supernatant was quantified in order to compare equal amounts of protein extract in western blotting. The samples were subjected to electrophoresis in 10% gels, blotted onto nitrocellulose membranes, and probed with primary antibody at 4 °C overnight. The following antibodies were used: anti-β-actin (A5316, Sigma–Aldrich), anti-CPT1b (22170-1-AP, Proteintech), anti-SREBP1 (AB3259, Abcam), and anti-SREBP2 (AB30682, Abcam). After washes, membranes were incubated with horseradish peroxidase-conjugated anti-mouse (7076, Cell Signaling Technology) or anti-rabbit (7074, Cell Signaling Technology) IgG for 1 h. The chemiluminescent signals were detected using an ECL system (LuminataTM Crescendo, Millipore). β-actin was used as loading control.

### 2.7. Cell Transfection

For transient transfection experiments, H9c2 cells were seeded in 6-well plates without antibiotics. The next day, antimiR, premiR, or siRNAs were transfected into cells according to previously reported procedures [21,24]. AntimiR and premiR transfections were performed in order to modulate miR-33a level. AntimiR-33a inhibitor is a single-stranded nucleic acid designed to specifically bind to and inhibit endogenous miR-33 molecules. PremiRNA-33a is a small, chemically-modified double-stranded RNA designed to mimic endogenous mature miR-33a. Ambion^®^ Anti-miR™ miRNA inhibitors, negative control (antimiR-NC) and antimiR-33a, and Ambion^®^ Pre-miR™ miRNA precursors, negative control (premiR-NC) and premiR-33a (Life Technologies), were transfected into cells at 50 nM concentration. For transient transfection experiments of SREBPs, cells were transfected with 25 nM ON-TARGETplus Rat SREBP1 and/or SREBP2 siRNA, or ON-TARGETplus non-targeting pool (Dharmacon). All transfections were performed by using Lipofectamine^®^ RNAiMAX (Invitrogen) in Opti-MEM^®^ Medium for 24 h (Life Technologies). Next, cell medium was changed with or without FA supplementation and cells were incubated for 16 or 24 h. Finally the cells were collected for subsequent analysis.

### 2.8. Statistical Analysis

Data are presented as mean ± standard error of mean (SEM), of at least three independent determinations. All statistical analysis was performed using Prism 5 (GraphPad Software, La Jolla, CA, USA). Student’s t-test or one-way ANOVA with Newman–Keuls multiple comparison test was performed and a *p*-value lower than 0.05 was considered statistically significant. In particular, differences are indicated with ns (not significant) for a *p*-value > 0.05, or with *, **, ***, for *p* < 0.05, *p* < 0.01, and *p* < 0.001, respectively.

## 3. Results

### 3.1. EPA and DHA Prevent Apoptosis and Hypertrophy Induced by Palmitate in H9c2 Cardiac Cells

In a previous report we have shown that treatment of H9c2 cardiac cells with palmitate decreases cell viability in a time- and dose-dependent manner, with a maximal effect at 500 μM [20]. The loss of cell viability was due to apoptotic cell death and was prevented by co-treatment with EPA added at a concentration as low as 60 μM. Therefore, we have used these concentrations of palmitate and n-3 PUFA in all the experiments described in the present work.

**Figure 1:**
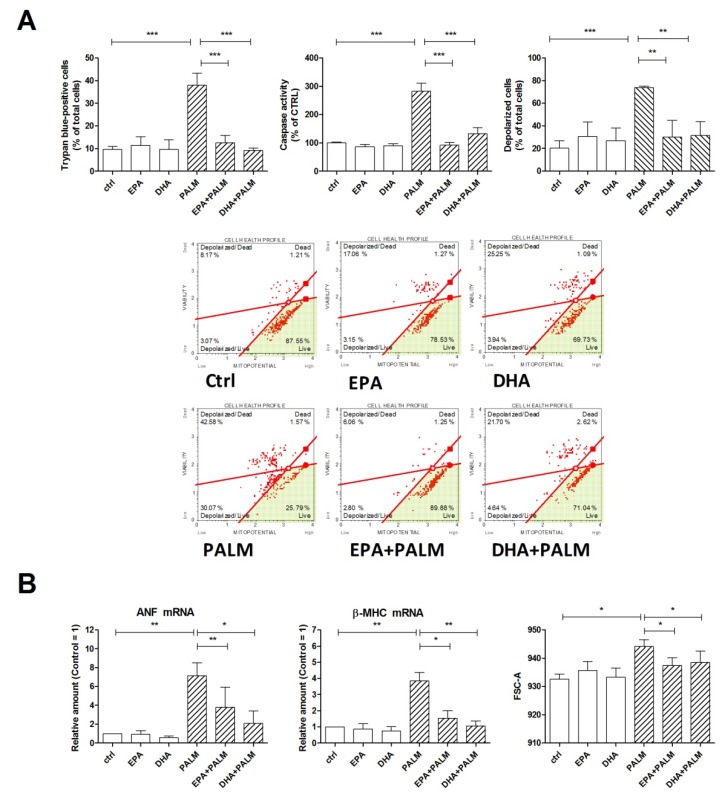
Effect of n-3 polyunsaturated fatty acids (PUFAs) on hypertrophy, mitochondrial potential, and survival of H9c2 cardiac cells exposed to palmitate. H9c2 cells were incubated under control condition (ctrl), in the presence of 500 µM palmitate (PALM), 60 μM eicosapentaenoic acid (EPA), 60 μM docosahexaenoic acid (DHA), or a combination of fatty acids, as indicated, (EPA+PALM; DHA + PALM). (**A**) Cell viability was assessed after 24 h treatment by trypan blue exclusion test to calculate the percentage of dead cells or analyzed for caspase activity. Alternatively, H9c2 cells were treated for 16 h, then analyzed for mitochondrial membrane potential to calculate the percentage of depolarized cells. Panel A, bottom: representative dot plot data output of mitochondrial depolarization are reported. (**B**) After a 16 h incubation, H9c2 cells were analyzed by RT-PCR for the relative amount of ANF mRNA and β-MHC mRNA, as markers of cardiac hypertrophy; in addition, cell size was analyzed in a flow cytometer by measuring the forward scattering (FSC). Differences are indicated with ns (not significant) for a *p*-value > 0.05, or with *, **, ***, for *p* < 0.05, *p* < 0.01, and *p* < 0.001, respectively.

Figure 1A shows that not only EPA, but also DHA exerted a protective effect on palmitate-induced cell death and caspase 3-like activation. Besides, palmitate provoked an early loss of mitochondrial membrane potential that was also prevented by co-treatment with EPA or DHA. It should be noted that the n-3 PUFAs alone did not modify significantly cell viability, caspase activity and mitochondrial potential at the same concentrationthat protected from palmitate. Thus, these results show that palmitate can cause an apoptotic cell death involving mitochondrial dysfunction, which can be prevented by co-treatment with substantially lower doses of long chain n-3 PUFAs.

Since a high fat diet is able to promote cardiac hypertrophy in rats and specifically palmitate can induce hypertrophy of H9c2 cells [2], we have also assessed the effect of palmitate, in the presence or absence of n-3 PUFAs, on the hypertrophic response of H9c2 cells, evaluated as an increase of cell size and enhanced expression of fetal genes, such as ANF and β-MHC. Figure 1B shows that palmitate markedly increased the mRNA levels of the hypertrophy-related genes, i.e., ANF and β-MHC; moreover, both EPA and DHA significantly reduced the expression of these genes when given together with palmitate. Finally Figure 1B depicts how palmitate significantly increased H9c2 cell size and this effect was prevented by co-incubation with EPA or DHA. Thus, we observed a similar protective pattern for the n-3 PUFAs against both apoptotic and hypertrophic effects of palmitate.

### 3.2. Effect of Palmitate, EPA, and DHA on the Expression of SREBP Genes and of Genes Encoding Enzymes Driving FA Fate

The SREBP family consists of transcription factors SREBP1a and 1c, produced from a single gene (*SREBF1*) through the use of alternative transcription start sites, and SREBP2 produced from a distinct gene (*SREBF2*) [11]. In their active form, SREBP proteins act as master transcription factors for the expression of enzymes mainly involved in lipogenesis and cholesterol biosynthesis.

**Figure 2:**
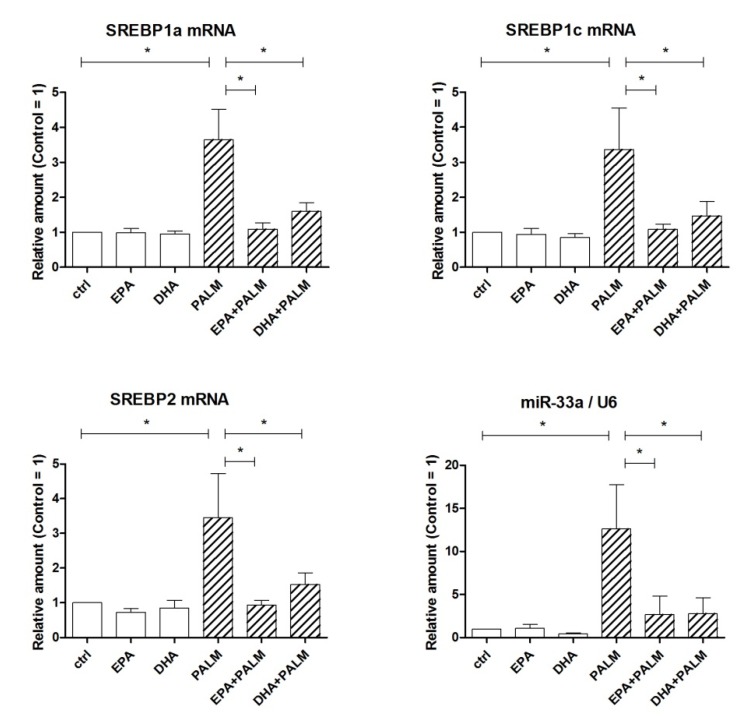
Effect of n-3 PUFAs on the expression of SREBP genes in H9c2 cells exposed to palmitate. H9c2 cells were incubated in the presence or absence of fatty acids as described in the legend of Figure 1. After a 16 h incubation, H9c2 cells were analyzed by RT-PCR for the relative amount of SREBP1a mRNA, SREBP1c mRNA, SREBP2 mRNA, and miR-33a. Differences are indicated with * for *p* < 0.05.

Figure 2 shows the effect of palmitate and/or each n-3 PUFA on the expression of SREBP mRNAs. Palmitate was able to significantly increase the mRNA levels of all three SREBPs, i.e., SREBP1a, 1c, and 2. All these increases were markedly reduced or prevented by co-incubation of the cells with EPA or DHA. MiR-33a, an intronic microRNA hold in the human and rat SREBP2 gene, was co-expressed along with the transcript of its host gene under the control of the same promoter and potentially under the same stimuli. Indeed, non-surprisingly we observed that miR-33a expression showed the same pattern following exposure of H9c2 cells to saturated and polyunsaturated FAs.

Long-chain acyl-CoA synthetase 1 (ACSL1) is an enzyme highly expressed in the heart catalyzing the activation of long chain FAs to acyl-CoA, a key step for further use of FAs [25,26]. Since EPA has been reported to suppress palmitate-induced expression of ACSL1 in macrophages, presumably via SREBP1a regulation [27], we have investigated the effect of palmitate and/or n-3 PUFAs on the expression of ACSL1 in H9c2 cells. We have also examined the gene expression of key enzymes that use acyl-CoA as a substrate thus deciding FA fate, i.e., diacylglycerol acyltransferase (DGAT) and serine palmitoyl-transferase (SPTLC). Unrelated genes encode the two enzymes DGAT1 and DGAT2, which have both overlapping and different properties. The first one is active in esterifying exogenous FAs taken up by cells and has been reported to act even as a monoacylglycerol acyltransferase (MGAT) thus allowing the synthesis of DAG [28]. DGAT2 seems to exert the prominent role in mammalian TG synthesis [29]. SPTLC is an enzymatic complex comprising subunits 1, 2, and 3, which catalyzes the first and rate-limiting step in the de novo biosynthesis of ceramide by condensing serine and palmitoyl-CoA [5,30].

**Figure 3:**
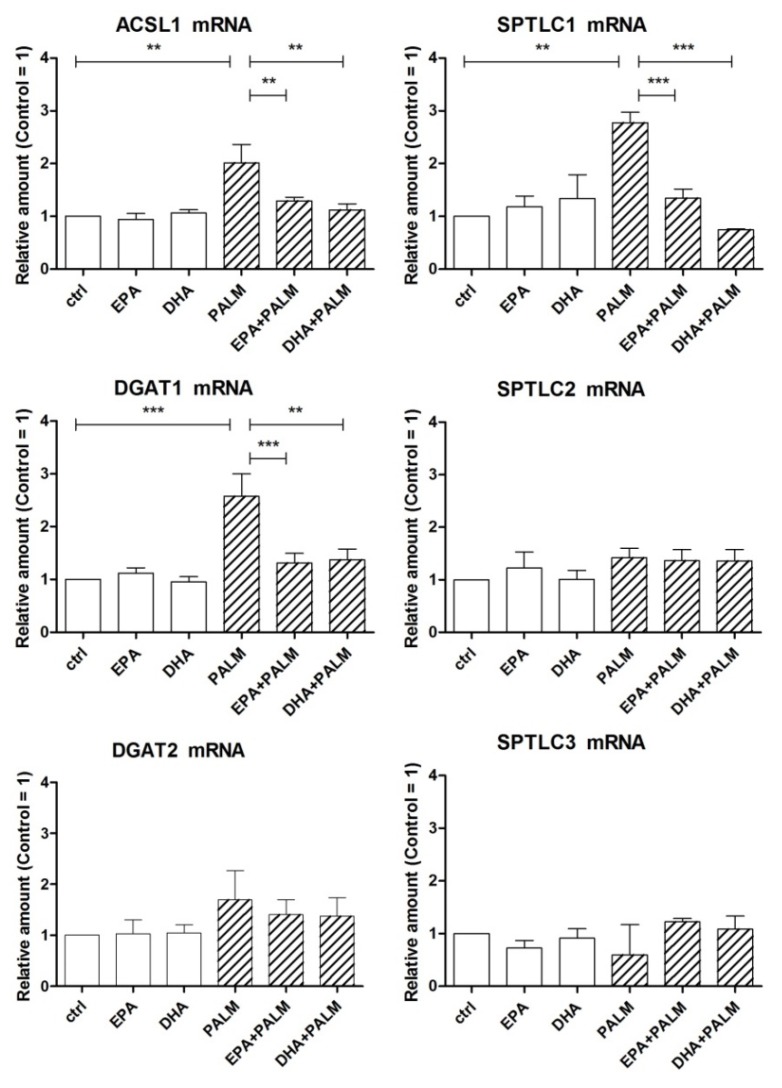
Effect of n-3 PUFAs on the expression of genes coding for key enzymes related to fatty acid metabolism in H9c2 cells exposed to palmitate. H9c2 cells were incubated in the presence or absence of fatty acids as described in the legend of Figure 1. After a 16 h incubation, H9c2 cells were analyzed by RT-PCR for the relative amount of ACSL1 mRNA, DGAT1 mRNA, DGAT2 mRNA, and SPTLC subunit mRNAs. Differences are indicated with ** or *** for *p* < 0.01 and *p* < 0.001, respectively.

As shown in Figure 3, palmitate significantly increased the level of ACSL1 mRNA in H9c2 cells, whereas EPA or DHA even prevented this palmitate effect. Similar patterns were obtained for DGAT1 and SPTLC1 mRNA, whereas no significant modulation of gene expression was observed for DGAT2, SPTLC2, and SPTLC3.

**Figure 4:**
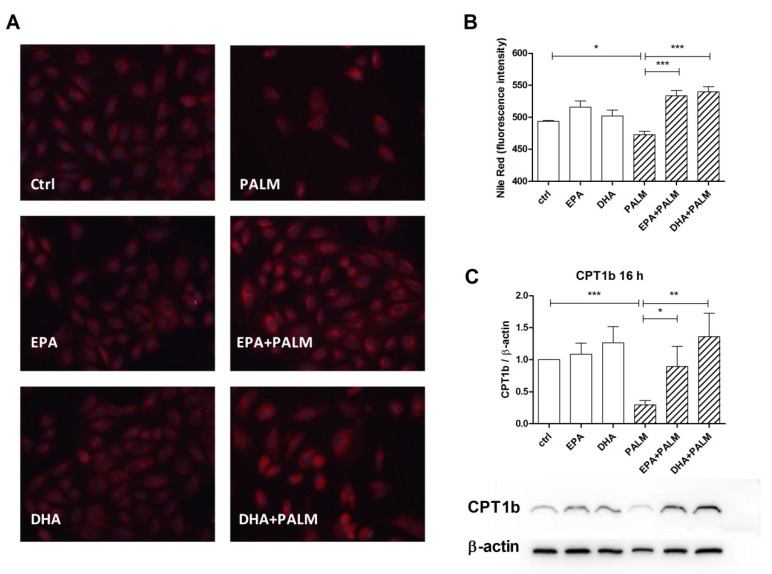
Effect of n-3 PUFAs on the accumulation of neutral lipids and the expression of CPT1b protein in H9c2 cells exposed to palmitate. H9c2 cells were incubated in the presence or absence of fatty acids as described in the legend of Figure 1. After a 16 h incubation, H9c2 cells were analyzed for the content of neutral lipids by Nile red staining or for CPT1b protein content. (**A**) Representative microscopy fields of cells stained with Nile red. Scale bar corresponds to 50 µm. (**B**) Quantitative analysis of Nile Red fluorescence by flow cytometry. (**C**) Relative quantification for CPT1b/β-actin ratio by western blot. Panel C, bottom, shows a representative blot image for CPT1b and β-actin. Differences are indicated with *, **, *** for *p* < 0.05, *p* < 0.01 and *p* < 0.001, respectively.

These results suggest that the treatment with palmitate can favor the metabolic activation of fatty acids and their addressing towards DAG, TG, or ceramide biosynthesis. However, the accumulation of neutral lipids in H9c2 cells was slightly, but significantly reduced following palmitate supplementation, and actually increased or restored by n-3 PUFAs (Figure 4A,B). In particular Figure 4B reports the quantitative analysis of the fluorescence intensity detected in single cells by flow cytometry.

We have also evaluated the possible modulation of the level of CPT1b protein by FAs, since this enzyme, which limits the rate of long-chain fatty acids translocation to the mitochondrial matrix to be β-oxidized, is considered a target of miR-33 [14]. Figure 4C shows that palmitate addition markedly decreased, while co-administration of EPA and DHA restored the CPT1b level. Thus, modulation of the content of this protein varied in an opposite way with respect to that of miR-33a shown in Figure 3.

### 3.3. Effects of Manipulating the Expression of miR-33a and SREBPs on Cell Death and Hypertrophy

Since the results described above showed that the expression of miR-33a and SREBPs markedly increased following palmitate supplementation, but not after n-3 PUFA, we have investigated their role in cell death and hypertrophy of H9c2 cells by silencing their expression.

**Figure 5:**
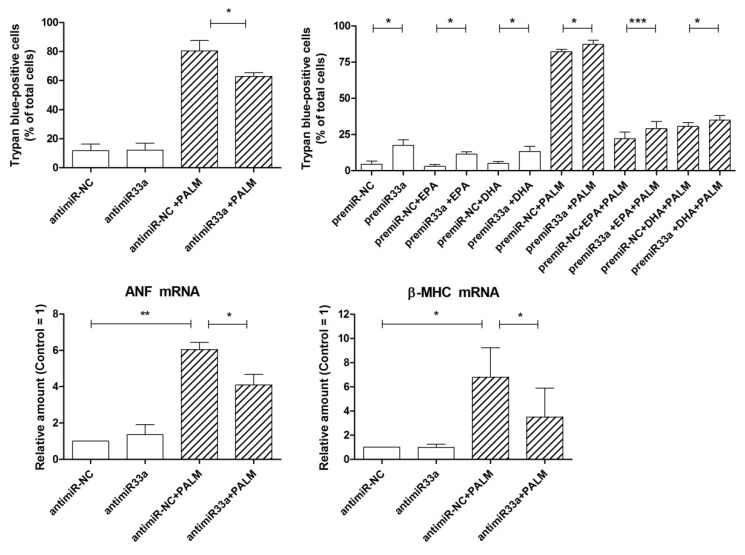
Effect of miR-33a on survival and hypertrophy of H9c2 cardiac cells exposed to palmitate. **Top**: cells were transfected for 24 h with antimiR-33a or antimiR-NC (50 nM) and premiR-33a or premiR-NC (50 nM). After a 24 h incubation with or without fatty acids, as indicated, cells were counted to assess cell viability by trypan blue exclusion test. **Bottom**: cells were transfected for 24 h with antimiR-33a or antimiR-NC (50 nM). After a 16 h incubation with or without palmitate, H9c2 cells were analyzed by RT-PCR for the relative amount of ANF mRNA and β-MHC mRNA, as markers of cardiac hypertrophy. Differences are indicated with *, **, *** for *p* < 0.05, *p* < 0.01 and *p* < 0.001, respectively.

Figure 5 (top) shows that the transfection of cells with antimiR-33a actually reduced the number of dead cells following palmitate with respect to a proper control by about 20%. Conversely, the treatment with premiR-33a enhanced the number of dead cells to almost 20% of total cells in the absence of palmitate and slightly increased cell death even after palmitate addition. It can also be noted that similar results were obtained in the presence of n-3 PUFA, either EPA or DHA. Furthermore, Figure 5 (bottom) shows that antimiR-33a transfection significantly reduced the increase of the palmitate-stimulated expression of the markers of cardiac hypertrophy, ANF, and β-MHC, by about 40–60%. Therefore, it may be deduced that miR-33a is involved in a limited manner in the palmitate-induced loss of viability of H9c2 cells and plays a more important role in the development of hypertrophy of these cardiac cells.

**Figure 6:**
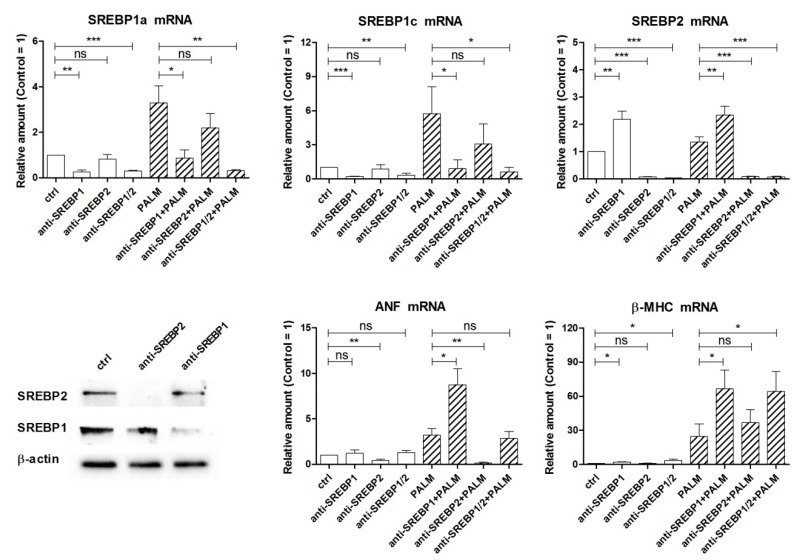
Effect of SREBP siRNAs on SREBP mRNA levels and hypertrophy of H9c2 cardiac cells exposed to palmitate. Cells were transfected for 24 h with SREBP1 siRNA and/or SREBP2 siRNA (indicated as anti-SREBP in the figures). After a 24 h incubation with or without palmitate, as indicated, cells were analyzed by RT-PCR for the relative amount of SREBP1a, SREBP1c, SREBP2, ANF, and β-MHC mRNAs. Western blot of SREBP1 and SREBP2 is reported to show the effectiveness of silencing. Differences are indicated with ns (not significant) for a *p*-value > 0.05, or with *, **, ***, for *p* < 0.05, *p* < 0.01, and *p* < 0.001, respectively.

Figure 6 shows the results of silencing the expression of SREBPs under basal conditions or after palmitate induction, by using SREBP1 and/or SREBP2 siRNA. First, the effects on the levels of the mRNAs of SREBP1a, SREBP1c, and SREBP2 were examined. Transfection of H9c2 cells with SREBP1 siRNA markedly reduced the messenger levels of both SREBP1a and SREBP1c, confirming the efficacy of silencing, and significantly increased the amount of SREBP2 mRNA both in the absence and presence of palmitate. Moreover, transfection with SREBP2 siRNA markedly reduced the level of SREBP2 mRNA, as expected, but exerted no significant effect on the amount of SREBP1a and SREBP1c messengers, both under basal and palmitate-stimulated conditions. Finally, a significant and remarkable reduction of all three mRNAs was observed when H9c2 cells were treated simultaneously with both siRNAs. These results indicate that in our experimental model the relationship between SREBPs is unidirectional, in that SREBP1 down-regulation affects SREBP2 expression, but not vice versa, in accordance with early findings with SREBP knockout mice. In particular, SREBP1^–/–^ mice lacking both the 1a and 1c transcripts exhibit an increase in hepatic SREBP2 mRNA and protein, which in turn leads to increased cholesterol biosynthesis and also partially compensates for the lack of SREBP1 isoforms in lipogenesis [9,10].

Next we evaluated the effects of knocking down SREBPs expression on cell viability by trypan blue test, but we could not observe any significant difference in the percentage of dead cells between control cells and cells treated with SREBP1 and SREBP2 siRNAs, either alone or in combination (data not shown). In the end, we have examined the effects of these siRNAs on the expression of ANF and β-MHC. SREBP2 siRNA reduced the basal level of ANF mRNA and completely prevented its induction by palmitate, while it did not modify the amount of β-MHC mRNA significantly. Instead anti-SREBP1 treatment proved to be able to increase the levels of both ANF and β-MHC mRNAs, in cells incubated with palmitate (and β-MHC mRNA also in cells without palmitate). The latter effect may occur via enhanced SREBP2 gene expression in the case of ANF or be independent of this. Thus, these results indicate that SREBP2, differently from SREBP1a and/or SREBP1c, can stimulate ANF expression and may contribute to the induction of cell hypertrophy in H9c2 cells.

**Figure 7:**
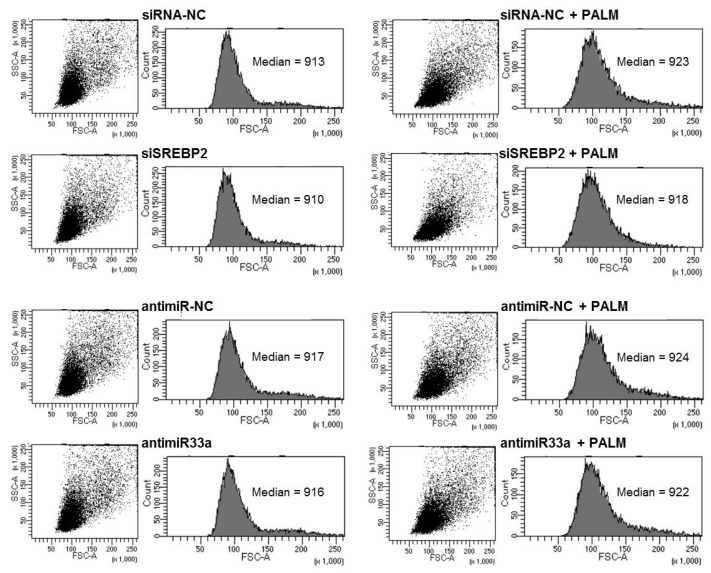
Effect of SREBP2 and miR33a silencing on cell size after palmitate treatment. H9c2 cells were transfected for 24 h with SREBP2 siRNA or antimiR33a. After 16 h of incubation with or without palmitate, cells were fixed and analyzed in a flow cytometer by measuring the forward scattering (FSC).

In addition, we assessed the effect of SREBP2 and miR-33a silencing on cellular size by flow cytometry. In accordance with data obtained from gene expression evaluation, silencing of either SREBP2 or miR-33a seems to reduce palmitate-induced hypertrophy, as shown in Figure 7.

## 4. Discussion

First, the present study confirms and extends our previous finding of a protective effect of EPA in H9c2 cardiac cells supplemented with a relatively high dose of palmitate [20]. In fact, we show here that not only EPA, but also DHA, exerted a preventive action versus palmitate-induced cell death and apoptosis, which appeared to be preceded by a loss of mitochondrial membrane potential. Besides, both long-chain n-3 PUFAs were able to impede the induction of heart hypertrophy markers and the increase of cell size elicited by palmitate. Other studies have shown that addition of palmitate in excess can favor cell death and even hypertrophy of cardiac cells [2,31]. In addition, n-3 PUFAs have been reported to exert protective actions, in particular, EPA attenuated endothelin-induced hypertrophy of neonatal cardiomyocytes [32] and ALA protected against isoproterenol-induced reduction of viability of H9c2 cells and cardiac hypertrophy in rats [33]. A recent study concluded that linoleic acid, an n-6 PUFA, is a potent inducer of genes involved in inflammatory and hypertrophic responses of adult rat cardiomyocytes, even more than palmitic acid, whereas DHA can decrease the expression of genes associated to cardiac hypertrophy, such as ANF and BNF [34]. However, the molecular mechanisms underlying the actions of FAs on cardiac cell viability and growth are not clearly defined.

In the current work we have evaluated the effect of palmitate on key enzymes involved in driving its metabolic fate and found that palmitate increases the expression of ACSL1 and SPTLC1. This may favor metabolic activation of palmitate to palmitoyl-CoA and subsequent synthesis of ceramide, a mediator of cardiac lipotoxicity [5,25]. It has been proposed that DGAT1 overexpression ameliorates heart lipotoxicity in some situations by diverting FAs from potential cytotoxic fates, such as ceramide and DAG, to inert TG stored in lipid droplets [8]. Although DGAT1 expression was increased by palmitate addition in our experimental model, accumulation of neutral lipids did not increase and actually was found to be lower with respect to cells supplemented with an n-3 PUFA. This finding may be in accordance with some studies showing that palmitate-mediated lipotoxicity in neonatal cardiomyocytes as well as in AC16 and H9c2 cardiac cell lines was associated with impaired formation of lipid droplets and stress of endoplasmic reticulum (ER), due to packaging of palmitate primarily as DAG in ER, rather than as TG in lipid depots [35,36]. Instead EPA and DHA supplementation was found to lead to increased TG storage [37].

Addressing of palmitate towards ceramide and DAG synthesis may also be facilitated by the loss of CPT1b, thus by the prevented entry and oxidation of palmitate in heart mitochondria. Interestingly heterozygous CPT1b knockout mice were more prone to cardiac hypertrophy and exhibited mitochondrial abnormalities and ceramide accumulation, leading to cardiomyocyte apoptosis [38]. Conversely, increased mitochondrial FA oxidation by expression of a mutant form of CPT1, insensitive to malonyl-CoA inhibition, has been shown to protect skeletal muscle cells from palmitate-induced apoptosis and insulin resistance [39]. Impaired mitochondrial FA uptake and oxidation has also been associated to lipotoxicity in cultured cardiac cells, that may occur through early events that are independent of CPT1 down-regulation [40].

A main finding of our study is the induction of miR-33a by palmitate treatment of H9c2 cardiac cells and the ability of n-3 PUFAs to counteract even this effect of palmitate. This pattern was opposite to that showed by CPT1b protein; besides, premiR-33a transfection resulted in down-regulation of CTP1b (data not shown), which is consistent with CPT1 being a direct target of miR-33. Chen et al. [14] have reported that this microRNA specifically regulates myocardial β-oxidation in mouse, rat, and human cardiac cells and in particular mediates the inhibition of FA oxidation by TXNIP, a protein that has emerged as an important player in myocardial pathology, promoting oxidative stress as well as cardiomyocyte apoptosis and hypertrophy. Manipulation of miR-33a levels actually affected the viability of H9c2 cells, even if to a limited extent, thus indicating that the induction of this microRNA by palmitate can only moderately contribute to cell death. More critical appears to be the role of miR-33a in the development of H9c2 cell hypertrophy, since antimiR-33a transfection more markedly reduced the expression of hypertrophy marker genes, ANF and β-MHC. Mir-33 is a highly-conserved microRNA that is considered a potential target for atherosclerosis, since it has pro-atherogenic effects by directly targeting ABCA1 and ABCG1 cholesterol transporters, thus lowering HDL-cholesterol, and is involved in inflammatory processes and ER stress in atherosclerosis lesions [41,42]. In addition, miR-33 targets insulin receptor substrate 2 (IRS-2) and reduces insulin signaling in the liver [15]. However, more recently, miR-33 has also been involved in cardiac remodeling [43] and in coronary heart disease [44]. Our results suggest that enhanced miR-33a expression can contribute to the responses of cardiac cells to elevated palmitate exposure and even to high-fat diet in vivo, together with other microRNAs, such as miR-451, which has been reported to increase in palmitic acid-stimulated neonatal rat cardiomyocytes [3]. In this context, a possible role of miR-33 in palmitate-induced insulin resistance of cardiac cells remains to be investigated.

In our cell model, palmitate supplementation induced the expression of SREBP2 gene and miR-33a, as well as the expression of SREBP1 gene, codifying for SREBP1a and SREBP1c mRNAs. To our knowledge the present paper is the first showing the ability of n-3 PUFAs to inhibit the up-regulation of both SREBP1 and 2 genes by palmitate in cardiac cells. It is known that palmitate can induce SREBP1c in liver and in some cell types, whereas n-3 PUFAs down-regulate its expression and maturation in some contexts [10,11,45,46]. In particular, exposure of pancreatic β-cells to palmitate provoked SREBP1c induction, while treatment with a SREBP1 siRNA or reduced SREBP1 expression by exendin-4 or metformin exerted protective effects against palmitate-induced β-cell dysfunction and apoptosis [47,48]. SiRNA experiments in H9c2 cells indicated that SREBP factors do not improve cell viability but may contribute to the control of cardiac cell hypertrophy. In fact, SREBP2 resulted to being required for the expression of ANF gene, a marker of cardiac hypertrophy, instead SREBP1s appear to have an opposite effect, since their suppression by SREBP1 siRNA increased the expression of fetal genes. These findings suggest that SREBFs may be multitasking genes involved not only in the regulation of heart lipidic metabolism but also in cardiac remodeling. Expression of SREBP1 was found to be increased in fibronectin-stimulated cardiomyocytes, but its effective role in this cellular model of cardiac hypertrophy was not investigated [49]. Interestingly, high levels of myocardial SREBP1 have been reported in patients with pressure overloaded heart and metabolic syndrome [50] and might be causally related to selective insulin resistance and inhibition of uncoupling protein 3 (UCP3) expression in the mouse heart [51]. Besides, the high expression of both SREBP1 and SREBP2 mRNA in epicardial adipose tissue has been identified as a cardiovascular risk factor in patients with type-2 diabetes [52]. Myocardial SREBP1c is also involved in the regulation of cardiac parasympathetic response [11].

## 5. Conclusions

In conclusion the present study highlights new aspects of fatty acid-related genetic and epigenetic regulation, elicited in H9c2 cells by high levels of palmitate and contrasted by lower concentrations of two different n-3 PUFAs, thus protecting cardiac cells from potentially negative outcomes. EPA and DHA appeared to be equally effective in our experimental model. The results here reported may have implications for the control of cardiac remodeling and dysfunction, particularly in relation to dietary habits and metabolic syndrome, and prompt further investigation on the role of *SREBF* gene products in the cardiac cell responses to saturated and polyunsaturated FAs.

## Figures and Tables

**Table 1:** Real-Time RT-PCR primers

Gene	Forward	Reverse
GAPDH	GACCTCAACTACATGGTCTACA	ACTCCACGACATACTCAGCAC
SREBF1A	CCATGGACGAGCTACCCTTC	GGCACTGGCTCCTCTTTGAT
SREBF1C	GATTGCACATTTGAAGACATGC	GCACGGACGGGTACATCTTTA
SREBF2	AGCACACTTGTCGAGATCCA	CCTTGGCTGCTGACTTGATC
ANF	TGGGCTCCTTCTCCATCACC	GCCAAAAGGCCAGGAAGAGG
β-MHC	GCCTACCTCATGGGACTGAA	ACATTCTGCCCTTTGGTGAC
DGAT1	CCCATACCCGGGACAAAGAC	AGAGTCTTGCAGACGATGGC
DGAT2	CGTGAGGCGGCTTCCTG	GAGGATGCTGGAGCCAGTG
SPTLC1	GAGGGTACGGGGATGAGTCT	TGAGCAAGCGGCTATCCAAA
SPTLC2	CTCTACATGCCGGCCAAAAT	TCAAAGCCGTGTCAAGTATTTCT
SPTLC3	CCAAGGCATCCGAGAGTTGT	AGCACTATGCGACTGAACCC
